# Effectiveness of Tamaru method plasma therapy: Overview and the original method

**DOI:** 10.3892/mi.2026.339

**Published:** 2026-10-08

**Authors:** Hiroyuki Ono, Takuji Yamaguchi, Ailing Hu, Naoto Hayashida, Hisashi Takeuchi

**Affiliations:** 1 ATP Salus Laboratory Inc., Tokyo 107-0052, Japan; 2 Department of Personalized Kampo Medicine, Juntendo University Graduate School of Medicine, Tokyo 113-8421, Japan; 3 Department of Preventive and Community Dentistry, Nippon Dental University, Tokyo 102-8159, Japan; 4 Japan Society of Advanced Medical Sciences, Tokyo 151-0063, Japan

**Keywords:** non-equilibrium atmospheric pressure plasma, plasma oncology, non-invasive therapy, reactive oxygen species, adenosine triphosphate, nitric oxide, elderly cancer care

## Abstract

The rapid aging of the population in Japan has led to an increased incidence of cancer, creating a demand for effective therapeutic options with minimal physical and financial burden. Non-equilibrium atmospheric pressure plasma (NEAPP), or low-temperature plasma, has emerged as a promising modality in oncology. The present study summarizes the fundamental principles of NEAPP and its therapeutic applications in cancer treatment. The authors first provide an overview of established approaches, such as the direct irradiation of cancer cells and the indirect use of plasma-activated medium, which have been shown to induce apoptosis in malignant cells. Subsequently, the authors introduce a novel therapeutic system, the Tamaru Method Plasma Therapy, developed by the authors' research group. This system combines a unique plasma-generating device, termed Plasma AIAS, with the oral intake of plasma-activated water. The proposed mechanism of this non-invasive method is the systemic enhancement of intracellular adenosine triphosphate production and the generation of nitric oxide. This dual action is expected to improve microcirculation and exert anti-tumor effects, while maintaining a high safety profile. The present study highlights the potential of this original method as a next-generation therapy for cancer, addressing the needs of modern medical care.

## Introduction

Japan is experiencing accelerated population aging, with the proportion of individuals aged ≥65 years projected to increase from 28.6% in 2020 to 38.7% in 2070(1). Cancer, which often results from the accumulation of genetic abnormalities in cells, is a disease prevalent among the elderly, and its incidence is expected to rise concurrently with this demographic shift (2). Furthermore, a significant number of cancer patients, ~23.9% (236,603 individuals) in 2021, are of working age (25-64 years) (3). Advances in medical check-ups and treatments have transformed cancer from an immediately life-threatening illness into a manageable condition, allowing numerous patients to continue working during therapy. However, this creates challenges related to financial costs and work-life balance. Consequently, there is a pressing need for effective, affordable and minimally invasive treatments with fewer side-effects. The present study introduces a novel therapy developed by the author HO, with medical insights provided by medical researchers, to address these needs.

## Principles of low-temperature plasma for medical applications

Plasma is an ionized gas composed of ions, electrons and neutral particles, often referred to as the fourth state of matter. It is formed when a gas is energized, causing its atoms or molecules to ionize (4). Non-equilibrium atmospheric pressure plasma (NEAPP), also known as low-temperature plasma, can be generated at atmospheric pressure (10^5^ Pa). This property renders it suitable for direct application to biological systems, including in aqueous environments, without requiring a vacuum chamber (4).

Gas-discharge plasmas are broadly categorized into thermal equilibrium plasmas (high-temperature) and non-equilibrium plasmas (low-temperature). The generation of NEAPP requires the suppression of Joule heating, which can be achieved by methods, such as limiting the current, using dielectric barriers, or applying pulsed voltages (5). The high-energy electrons in NEAPP excite or dissociate surrounding neutral molecules, generating reactive species. The key advantages of NEAPP include its ease of generation without a vacuum system and its high density of reactive species, which leads to rapid reaction rates (5).

The medical application of NEAPP has been an active area of research. In Japan, a large-scale academic project from 2012 to 2017, the ‘Creation of Plasma Medical Science’, significantly advanced its foundational science (6), with the findings compiled in ‘Plasma Medical Science’ (7). Cancer is characterized by the uncontrolled proliferation of cells that have evaded apoptosis (8). Early research in plasma oncology focused on the direct application of plasma to tumors to kill cancer cells (9). Methods include plasma jets, which deliver ions and radicals to the target site, and dielectric barrier discharge (10-12). Research has also demonstrated that plasma can selectively induce the apoptosis of cancer cells (13,14) and stimulate an antitumor immune response (15,16), representing indirect modes of action.

## Plasma-activated medium as an indirect therapeutic strategy

In 2011, Tanaka *et al* (17) reported that a cell culture medium exposed to NEAPP, which they named plasma-activated medium (PAM), induced the apoptosis of glioblastoma cells, demonstrating an antitumor effect. This discovery opened a new avenue for indirect plasma therapy. Subsequent studies demonstrated that PAM inhibits the proliferation of ovarian cancer cells (18) and suppresses peritoneal metastasis from gastric cancer (19). Numerous reports from research groups worldwide have since confirmed the antitumor effects of PAM (20-23). The mechanism is partly attributed to the induction of reactive oxygen species (24), which downregulate survival signaling pathways, such as AKT and ERK1/2 (MAPK) in cancer cells (25). Sensitivity to PAM can vary between cell types, and the precise intracellular mechanisms remain under investigation (26,27). Research has also expanded to other plasma-activated solutions, such as plasma-activated Ringer's lactate (PAL), which has been shown to exert antitumor effects through different, non-oxidative stress-dependent pathways (28,29). The clinical application of these solutions is an area of active research (30,31).

## The Tamaru method plasma therapy

The therapy that the authors are advancing, which is referred to as the Tamaru Method Plasma Therapy, combines treatment using a device known as Plasma AIAS (or Plasma Pulsar) with the oral intake of plasma-activated water.

This method is based on the ‘ATP enhancement device’, a technology developed and patented by Dr Shigeru Tamaru (32). The device itself, named ‘Plasma AIAS’, is designed to increase intracellular adenosine triphosphate (ATP) levels. ATP, discovered and elucidated by Dr Boyer, who received the Nobel Prize in 1997(33), is known as the ‘energy currency’ of life. It powers essential cellular processes through its hydrolysis to ADP (34,35). While the human body contains only a small amount of ATP, it recycles an amount equivalent to its body weight daily (34). Studies have suggested that restoring ATP levels may have therapeutic benefits, for instance by preventing protein aggregation (36-38), and this may be a strategy for the treatment of various diseases (39).

The Plasma AIAS device generates a plasma discharge by applying high-frequency, high-voltage pulses to water. When an electrode pad from the device is applied to a local area of the body, it is proposed that 2.5 million electrons per cm^2^ are delivered to the nerves at a rate of 3,000 times per second (40). The resulting intermittent electromagnetic field in the body of the user generates free electrons on the vascular walls, leading to the production of nitric oxide (NO). NO is known to increase capillary blood flow velocity, raise body temperature, and enhance ATP production (32). Furthermore, air from the device outlet contains a high concentration of free electrons. Inhaling this air is thought to cause the generation of NO on the pulmonary vascular walls, enhancing blood flow and ATP levels (32).

The water inside the device is also processed into plasma-activated water, which exhibits an oxidation-reduction potential similar to that of healthy human body fluids and contains abundant free electrons, NO and hydroxyl radicals. Drinking this plasma-activated water is intended to further stimulate NO generation on vascular walls, leading to improved blood flow and increased ATP levels (32).

The role of NO is considered crucial. The discovery that NO is the endothelium-derived relaxing factor by Dr Furchgott and others earned them the Nobel Prize in 1996 (41-43). NO is synthesized from L-arginine by endothelial NO synthase in endothelial cells and activates soluble guanylate cyclase in smooth muscle cells, leading to vasodilation (44). As NO is poorly soluble in water, drinking plasma-activated water provides a direct route for its absorption through the digestive tract.

In summary, the Tamaru Method Plasma Therapy aims to promote the generation of NO and ATP by introducing electrons through the skin, lungs, and digestive tract. This is expected to provide benefits, such as improved blood circulation, anti-aging and anticancer effects.

Despite the promising potential of the Tamaru Method Plasma Therapy, the present study has certain limitations. While the proposed mechanism relies on the physiological benefits of systemic ATP and NO enhancement, large-scale clinical data specifically targeting various cancer stages are still accumulating. Additionally, although no adverse events have been observed to date, further longitudinal studies are necessary to rigorously evaluate long-term safety and potential side-effects, particularly among the elderly population with diverse comorbidities. Future research is thus warranted to focus on clinical trials to provide robust evidence for its efficacy as a standardized cancer treatment.

In conclusion, medical expenses in Japan are a key component of social security costs, projected to reach 43.4 trillion yen in fiscal year 2025 (45,46). The Tamaru Method Plasma Therapy that the authors are advancing is an affordable and non-invasive option with no reported side-effects to date, rendering it a ‘low-risk, high-return’ therapy. The authors believe that it may contribute to extending healthy lifespans for both patients and healthy individuals. The authors will continue to accumulate clinical experience and research data on this plasma therapy and report the findings in the future.

## Data Availability

Not applicable.
